# Temporal invariance of centrality correlations and hierarchical topology in evolving cross-shareholding networks in Japan (2001–2023)

**DOI:** 10.1371/journal.pone.0331561

**Published:** 2026-03-25

**Authors:** Shinichiro Tanabe, Takaaki Ohnishi

**Affiliations:** 1 Graduate School of Artificial Intelligence and Science, Rikkyo University, Tokyo, Japan; 2 The Canon Institute for Global Studies, Tokyo, Japan; Universidad de la Republica Facultad de Ciencias Economicas y de Administracion, URUGUAY

## Abstract

In this study, we investigate the long-term evolution of the hierarchical structure and the stability of centrality correlations within Japan’s cross-shareholding networks. Using a 23-year dataset (2001–2023) of all listed Japanese companies, we conducted network analysis of semi-annually constructed networks of industrial sectors based on strongly connected components. To examine hierarchical structures, centrality distributions, and their intercorrelations, we applied bow-tie decomposition, PageRank, and the Hypertext-Induced Topic Selection (HITS) algorithm. The largest strongly connected component (the core group) decreased over time, whereas the number of companies that held shares in core members without being held in return (IN) or that remained entirely disconnected increased. Correlations among network measures exhibited temporal stability. The in-strength was positively correlated with both PageRank (*p*_*a*_), computed from the original directed network, and an authority score, whereas the out-strength was positively correlated with the Reversed PageRank (*p*_*h*_) computed from the reversed network and the hub score. A negative correlation was observed between *p*_*a*_ and *p*_*h*_. The correlation between in-strength and out-strength shifted from negative to positive around 2010, suggesting stronger cross-shareholding ties among companies. Most industries exhibited network-measure correlations similar to those observed in the overall network. In contrast, transportation equipment showed no significant correlation between in-strength and the corresponding *p*_*a*_, which suggests that firms were less influenced despite holding significant inbound voting rights. The relative ranking of network measures across industries remained stable over time. Banks consistently ranked low for in-strength and high for out-strength. Although their *p*_*h*_ ranks remained high, their hub score ranks decreased. These findings, such as the declining influence of banks and the rising centrality of the information & communication and real estate sectors, suggest that traditional firm-level or short-term monitoring may overlook systemic ownership structures. Periodic network-based monitoring can help to identify resilient and structurally influential firms or clusters.

## Introduction

Network science has contributed to our understanding of economic phenomena such as supply chains and stock markets [1,2]. The shareholding relationships of companies form complex structures characterized by loops or circular business relations [3]. Previous research has focused on multiple countries (macro), nations and regions, industries (meso), and corporate groups (micro). Differences in ownership structures across countries [4,5], the roles of financial institutions and index funds [6,7], government control [8,9], and hierarchical structures have also been explored in the literature [10].

Structural ownership ties such as cross-shareholding and ownership overlaps can significantly influence corporate behavior and market outcomes. These connections can also lead to increased risks of collusion in densely connected networks [11]. The interconnected nature of shareholding networks can facilitate cascading failures during economic shocks [12]. Conglomerates and business groups have long maintained extensive cross-ownership of shares in Japan to strengthen corporate ties and stabilize management [13]; this has also been observed in Germany, France, Spain, and South Korea [14–16]. High levels of cross-shareholding have been associated with overinvestment and low profitability [17], as well as with higher systemic risk [18,19]. For example, in China, the weakening of equity ties between the state and firms reflects a decline in direct government intervention [20], whereas the global petrochemical and food industries exhibit dense corporate networks that reinforce collective interests and policy influence [21,22]. Thus, both investors and policymakers have called for the dissolution of such arrangements [23]. In response to concerns about inefficiency and entrenchment, the Japanese government introduced the Corporate Governance Code in 2015 to promote transparency and reduce cross-shareholding [24]. Indeed, such ties have gradually declined due to economic crises, accounting reforms, and policy interventions [25].

However, the ways that this gradual decline has altered the overall network topology remain underexplored. Given that firm-level analyses may overlook systemic patterns, network-based measures of centrality can be applied to detect hierarchical or influential structures [26–29]. In this context, examining the statistical correlations among multiple measures of centrality is useful to assess whether they capture distinct or similar structural properties to help reveal the underlying topology and dynamics of corporate ownership networks [30–33].

Bow-tie decomposition is a method of directed network analysis in which a network is decomposed into components based on reachability to identify hierarchical structures [34,35]. Although this approach has been applied to stock ownership networks in Japan [10], to the best of our knowledge, it has not been applied to investigate long-term changes in cross-shareholding relationships in the relevant literature. If cross-shareholding has indeed declined, the core of the bow-tie structure is expected to have waned as well, and the degree and strength distributions may deviate from the power-law form observed in earlier studies [36]. However, to our knowledge this has not yet been empirically tested.

Although the PageRank algorithm was originally developed to rank pages on the web, it has been applied to various networks, including stock ownership networks [26–28]. The Hypertext-Induced Topic Selection (HITS) algorithm, which identifies authority and hub nodes, has also been used in the context of stock ownership to identify hub banks and financial institutions as key actors in ownership structures in the Italian market [29]. We adopted PageRank and HITS (authority and hub) scores as measures of centrality to quantify influence. Although PageRank tends to correlate with degree, it represents a global measure of influence, whereas authority and hub scores are influenced by two-step connections and are therefore not trivially correlated with degree [37]. In corporate ownership networks, multilayered structures such as parent, subsidiary, and sub-subsidiary relationships coexist, and affiliated firms often exhibit similar financial policies. Consequently, direct ownership alone does not capture the structure of influence. In such cases, indirect ownership ties have systemic significance because they can affect capital strategies and corporate decisions [38].

In this study, we analyzed the shareholding networks of all listed Japanese companies from 2001 to 2023 using bow-tie decomposition, PageRank, and HITS algorithms to systematically characterize changes in cross-shareholding structures and similarities across industries. This work is the first to investigate correlations among PageRank, authority, and hub scores in a stock ownership network over such an extended period, including in the COVID-19 era. Studies focusing on the impact of COVID-19 have mostly analyzed corporate groups and banks [28], and some have been limited to early analyses of the pandemic [39].

We considered the following primary research questions in the course of this study. (1) How have the topological structures of ownership networks in Japan evolved since the early 2000s in terms of measures such as the bow-tie structure and the distributions of degree and strength? (2) What are the relationships between different measures of network centrality (e.g., PageRank, authority, and hub scores) in identifying influential industries? (3) How stable are these structural characteristics over time, especially during exogenous shocks such as the COVID-19 pandemic?

Based on these questions, we propose the following hypotheses. (1) The core of the bow-tie structure has shrunk as a result of the dissolution of cross-shareholding ties, and the degree and strength distributions no longer follow a clear power-law scaling. (2) Before the dissolution, during the 2000s, PageRank, authority, and hub scores were highly correlated, but this correlation weakened as cross-shareholding ties declined. (3) Structural changes in the network occurred discontinuously in response to exogenous shocks such as financial crises, policy interventions, and the COVID-19 pandemic, with the magnitude and timing of these changes differing across industries.

This study makes the following contributions. (1) We analyzed the long-term temporal changes in the hierarchical structure of cross-shareholding networks and differences across industries in relation to exogenous events. (2) We shed new light on the stability of the network’s correlation structure over time and showed that industries can be characterized based on this structure.

## Materials and methods

### Data description

The data used in this study were provided by the Nikkei Economic Electronic Databank System (NEEDS) and include information on major shareholders of all publicly listed companies in Japan. In addition to the major shareholders listed in the securities reports, the top 30 shareholders identified by Nikkei’s independent research were recorded as information at two fiscal year-end points, including at the end of the fiscal year (the Full Year (F)) the end of an interim period (the Interim Year (I)). The data covered the period from the Interim Year 2001 to the Full Year 2023. Our analysis covered 4,157 issuing firms. The shareholders comprised a diverse set of entities, including companies, individuals, trust banks, and others. The total number of shareholders was 87,418. All listed companies were originally classified into 33 industry sectors based on the categories used by the Tokyo Stock Exchange (TSE), such as banks, chemicals, machinery, and services. However, sector classifications were not recorded for companies that have been delisted. A full list of these sectors is provided in Table 1.

**Table 1:** Time-averaged percentage of SCCN in each industry.

Industry	${\left\langle \text{Percentage}\right\rangle }_{t}$
Wholesale Trade	9.91
Chemicals	7.58
Machinery	7.45
Unknown	6.55
Construction	6.50
Electric Appliances	6.50
Banks	6.38
Foods	5.06
Information & Communication	4.46
Services	4.33
Transportation Equipment	3.16
Other Products	3.06
Metal Products	2.79
Land Transportation	2.50
Glass & Ceramics Products	2.30
Iron & Steel	2.24
Retail Trade	2.21
Real Estate	2.08
Textiles & Apparels	1.92
Precision Instruments	1.64
Pulp & Paper	1.45
Warehousing & Harbor Transportation	1.41
Nonferrous Metals	1.31
Other Financing Business	1.27
Pharmaceutical	1.21
Electric Power & Gas	1.11
Securities & Commodities Futures	0.99
Rubber Products	0.74
Fishery, Agriculture & Forestry	0.46
Marine Transportation	0.39
Oil & Coal Products	0.36
Mining	0.26
Insurance	0.24
Air Transportation	0.20

### Cross-shareholding network

**Fig 1:**
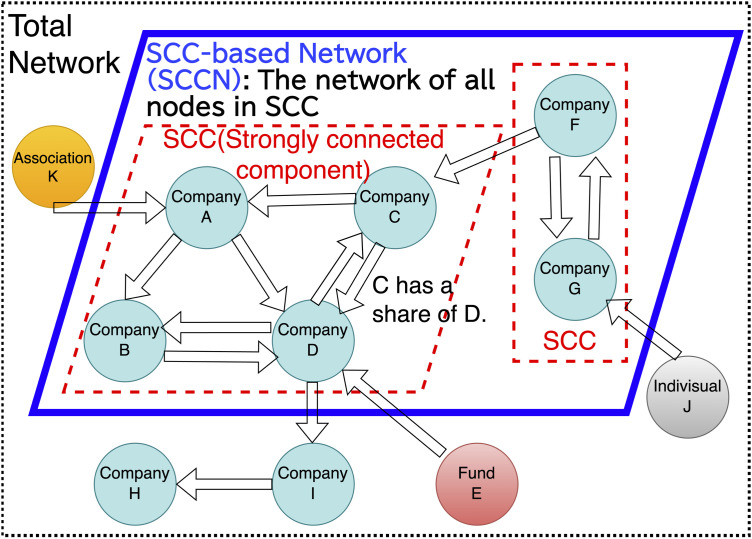
Schematic image of the construction of the stock ownership (total) network and cross-shareholding (SCC-based) network.

We constructed a weighted directed network to represent the shareholding relationships among companies. Each company was represented as a node *i*, and for each fiscal period, an edge (*i*,*j*) was drawn from company *i*, which owned shares, to company *j*, which was owned. The network was represented by a weighted adjacency matrix **A**, where each element *A*_*ij*_ denoted the weight of the directed edge from *i* to *j* corresponding to the percentage of voting rights held by *i* in *j*. An example of the constructed network is shown in Fig 1.

Because some companies use different fiscal months even within the same fiscal year, we utilized the interim and year-end reporting period provided by the data source. These flags identify the specific settlement month for each company’s mid-year (which we term “Interim Year”) and full-year (“Full Year”) financial settlements, respectively. This yielded 46 data points. The connections were modeled as a temporal network in which the numbers of nodes and edges changed over time. The size of the network *N* was defined by the number of nodes.

To examine cross-shareholding relationships, we analyzed a network derived from the original structure by removing nodes that did not belong to any strongly connected component (SCC) from the original network. In constructing this network, entities such as individuals with unidentified ultimate ownership, shareholding associations, venture capital firms, and trust accounts were excluded. We focused our analysis on this refined network, which we refer to as the SCC-based network (SCCN). The ratio of nodes belonging to each of 33 industry sectors, defined based on the Tokyo Stock Exchange classifications within the SCCN at each time point is provided in Table 1.

Table 2 summarizes the network statistics for the SCCN from 2001 to 2023. This table lists the number of nodes |V|, number of edges |E|, density *ρ*, average clustering coefficient ⟨C⟩, average path length *L*, and diameter *D*. The density is defined as follows.


$$\rho =\frac{\left|E\right|}{\left|V|(|V|-1\right)}.$$ (1)

**Table 2:** Summary of network statistics for the SCCN from 2001 to 2023. The number of nodes |V|, number of edges |E|, density *ρ*, average clustering coefficient ⟨C⟩, and diameter *D* are shown. “I” and “F” denote the reports issues at the end of the interim period and the full fiscal year, respectively (e.g., 2001I denotes the interim period of 2001).

Year	|V|	|E|	*ρ*	⟨C⟩	*D*	*L*
2001I	975	5854	0.0062	0.239	7	2.63
2001F	1051	7011	0.0064	0.231	7	2.62
2002I	1336	7351	0.0041	0.181	7	2.93
2002F	1348	8023	0.0044	0.227	7	2.69
2003I	1351	7291	0.0040	0.175	7	2.98
2003F	1396	7583	0.0039	0.178	8	3.00
2004I	1272	6358	0.0039	0.175	7	3.06
2004F	1284	6307	0.0038	0.170	8	3.10
2005I	1200	5828	0.0041	0.168	7	3.04
2005F	1206	6025	0.0041	0.177	6	3.00
2006I	1162	5319	0.0039	0.167	8	3.14
2006F	1142	5436	0.0042	0.174	7	3.06
2007I	1023	4291	0.0041	0.147	8	3.35
2007F	1140	4812	0.0037	0.146	7	3.27
2008I	1139	4681	0.0036	0.135	9	3.44
2008F	1185	4998	0.0036	0.140	8	3.39
2009I	1116	4749	0.0038	0.153	9	3.37
2009F	1184	5040	0.0036	0.138	7	3.41
2010I	1011	3691	0.0036	0.130	9	3.73
2010F	1016	3627	0.0035	0.133	9	3.82
2011I	908	2928	0.0036	0.124	9	4.10
2011F	932	3166	0.0036	0.133	11	4.00
2012I	883	2807	0.0036	0.129	10	4.12
2012F	913	3062	0.0037	0.132	9	3.97
2013I	876	2832	0.0037	0.127	13	4.15
2013F	888	2809	0.0036	0.134	10	4.15
2014I	811	2475	0.0038	0.134	10	4.23
2014F	820	2528	0.0038	0.137	10	4.21
2015I	768	2262	0.0038	0.132	11	4.38
2015F	779	2343	0.0039	0.137	10	4.29
2016I	739	2051	0.0038	0.126	11	4.59
2016F	715	2054	0.0040	0.132	11	4.48
2017I	732	2016	0.0038	0.116	11	4.57
2017F	742	2064	0.0038	0.118	12	4.57
2018I	655	1736	0.0041	0.127	13	4.78
2018F	681	1807	0.0039	0.124	13	4.77
2019I	686	1810	0.0039	0.135	12	4.76
2019F	681	1822	0.0039	0.129	12	4.72
2020I	671	1721	0.0038	0.127	14	4.90
2020F	687	1861	0.0039	0.129	12	4.64
2021I	723	1943	0.0037	0.102	14	4.74
2021F	673	1743	0.0039	0.120	16	4.84
2022I	698	1790	0.0037	0.097	12	5.12
2022F	731	2002	0.0038	0.112	13	4.69
2023I	622	1504	0.0039	0.103	14	5.29
2023F	563	1334	0.0042	0.086	15	5.49

The average clustering coefficient, computed on the SCCN converted to an undirected graph, is defined as


$$\left\langle C\right\rangle =\frac{1}{N}\sum_{i}C_{i},$$ (2)

where *C*_*i*_ is the clustering coefficient of node *i* given by


$$C_{i}=\frac{2t_{i}}{k_{i}(k_{i}-1)},$$ (3)

where *t*_*i*_ denotes the number of triangles that include node *i* and *k*_*i*_ denotes the degree of node *i*. The diameter is defined as the maximum shortest path distance between any two nodes in the network. This value was computed after converting the SCCN into an undirected graph and measured over the largest connected component. The average path length is defined as


$$L=\frac{1}{\left|R\right|}\sum_{(i,j)\in R}d(i,j),$$ (4)

where $R=\{(i,j)\in V^{\prime }\times V^{\prime }|i\neq j\}$ is the set of unordered node pairs, *V*’ denotes the set of nodes in the largest connected component of the SCCN after conversion to an undirected graph, and *d*(*i*, *j*) denotes the length of the shortest path between nodes *i* and *j*.

The number of nodes in the SCCN peaked at 1396 in the Full Year of 2003 and then decreased to 655 by the Interim Year of 2018. Subsequently, the number increased once again, reaching 731 by the Full Year of 2022, and then decreased again from the Interim Year of 2023. In the Full Year of 2023, the number of nodes was 563. The trend of changes in the number of edges was similar to that of nodes. The number of edges was 8023 in the Full Year of 2002. Although there were slight increases, such as from the Interim Year of 2001 to the Full Year of 2003 and from the Interim Year of 2008 to the Full Year of 2009, the overall trend was a steady decline. In the Full Year of 2023, the number of edges was 1334. The value of *ρ* remained low throughout the entire observation period, whereas ⟨C⟩ exhibited a decreasing trend, and both *D* and *L* showed increasing tendencies over time.

Although the SCCN is shrinking, the distribution of the size of each SCC in the SCCN remains similar across fiscal periods. SCCs other than the largest (LSCC) were significantly smaller than the LSCC [10]; in fact, the size of the LSCC was greater than 257, whereas the size of the second-largest SCC was less than 17.

### Bow-tie structure

**Fig 2:**
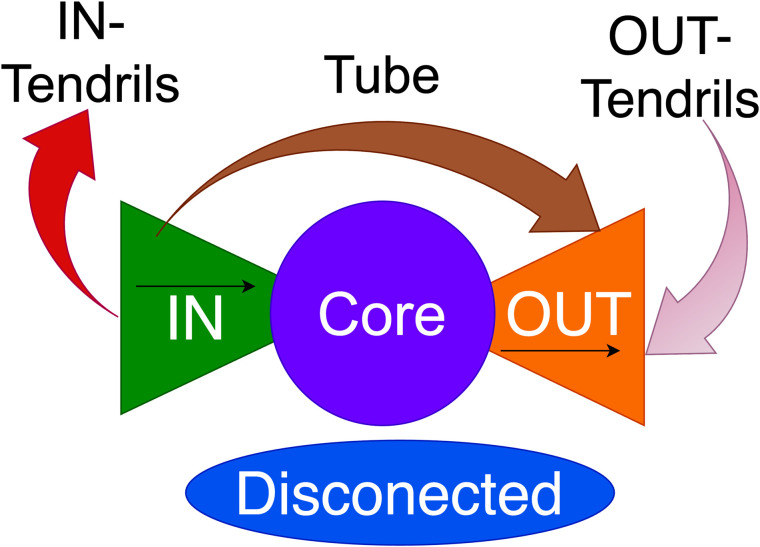
Schematic representation of the bow-tie structure.

We extracted hierarchical structures from directed networks using bow-tie decomposition [34,35]. Fig 2 shows a schematic representation of the bow-tie structure. Nodes are classified into components based on their reachability from other nodes via edges. The components of a bow-tie structure include the Core, IN Components (IN), OUT Components (OUT), IN-Tendrils, OUT-Tendrils, tubes, and disconnected nodes. The LSCC forms the Core. The nodes that can reach the Core but cannot be reached from it are IN, and those that can be reached from the Core but cannot reach it are OUT. Nodes that can be reached from IN but cannot reach the Core are IN-Tendrils, and those that can reach OUT but cannot be reached from the Core are OUT-Tendrils. Nodes that can be reached from IN and can also reach OUT without passing through the Core are Tubes, and nodes that do not belong to any of these components are classified as Disconnected. The hierarchical structure can be interpreted as IN being upstream, Core as midstream, and OUT as downstream. We first visualized the temporal changes in the bow-tie structure of the SCCN. Subsequently, we performed clustering to detect industries with similar temporal trends and identify similarities across industries.

### PageRank and HITS algorithm

The influence of individual companies on cross-shareholding relationships was measured using the PageRank and HITS algorithm. The PageRank of node *i*, $\hat{p_{i}}$, is defined as


$$\hat{p_{i}}=(1-\epsilon )\sum_{(j,i)\in V}\frac{\hat{p_{j}}}{out_{j}}+\frac{\epsilon }{N},$$ (5)

where ϵ=0.15, *V*, and *out*_*j*_ represent the damping factor, set of edges, and out-strength of node *j*, respectively [26]. The size of the network *N* is defined by the number of nodes. Because PageRank cannot be compared directly across networks with different numbers of nodes [40], we use a version of PageRank that is comparable with time series:


$$p_{i}=\frac{\hat{p_{i}}}{p_{low}}.$$ (6)

Here,


$$p_{low}=\frac{\epsilon +(1-\epsilon )\sum_{j\in D}\hat{p_{j}}}{N},$$ (7)

where *D* represents the set of nodes that only have in-strength and serves as a normalization to ensure that the lower limit of *p*_*i*_ is 1 [41].

Two types of PageRank can be computed from a single-directed network, including one from the original network and the other from a network in which all edge directions are reversed. The PageRank *p*_*a*_ calculated from the original network can be interpreted as susceptibility to influence from other companies. The Reversed PageRank *p*_*h*_ calculated by reversing the direction of the edges can be interpreted as the strength of its influence on other companies.

By applying the HITS algorithm, we identified authority and hub companies within cross-shareholding relationships. The HITS algorithm assigns authority and hub scores to each node in the network. A node’s authority score is increased if it receives many links from nodes with high hub scores. Similarly, the hub score increases when it points to numerous nodes with high authority scores. This recursive process defines the authority score *a*_*i*_ and hub score *h*_*i*_ of node *i* by using the following system of linear equations,


$$a_{i}=\sum_{j}A_{ji}h_{j},$$ (8)

$$h_{i}=\sum_{j}A_{ij}a_{j},$$ (9)

where the solution corresponds to the principal eigenvectors of the positive semidefinite matrices **A**^T^**A** and **AA**^T^, respectively. These are also the first right and left singular vectors of the matrix **A**. In this study, as in the PageRank computation, we replace *A*_*ij*_ with $(1-\epsilon )A_{ij}+\epsilon /N$, where ϵ=0.15.

**Fig 3:**
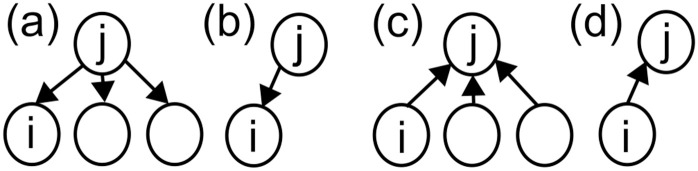
Example of link structures with high and low authority/hub scores. The authority score of node *i* increases when it shares shareholders with many other nodes (a) and decreases when it shares shareholders with fewer nodes (b). The hub score of node *i* increases when many other nodes are investing in the same companies as node *i* (c) and decreases when there are fewer such nodes (d).

In the unweighted case, a high authority score *a*_*i*_ indicates that the company has many shareholders, and that those shareholders also invest in many other firms (Fig 3(a), 3(b)). Industries with high authority scores can be interpreted as issuers of shares the investor bases of which include shareholders exerting a similar influence across multiple firms, potentially resulting in homogeneous corporate behavior within the industry. In contrast, company *i* has a high hub score *h*_*i*_ if it invests in many companies that, in turn, have many shareholders (Fig 3(c), 3(d)). Industries with high hub scores are characterized by intense competition among shareholders, which suggests that firms with low influence may have a limited impact on their investee companies. First, we visualized the distributions of *p*_*a*_, *p*_*h*_, *a* and *h* in the SCCN.

## Results

### Degree/strength distribution

**Fig 4:**
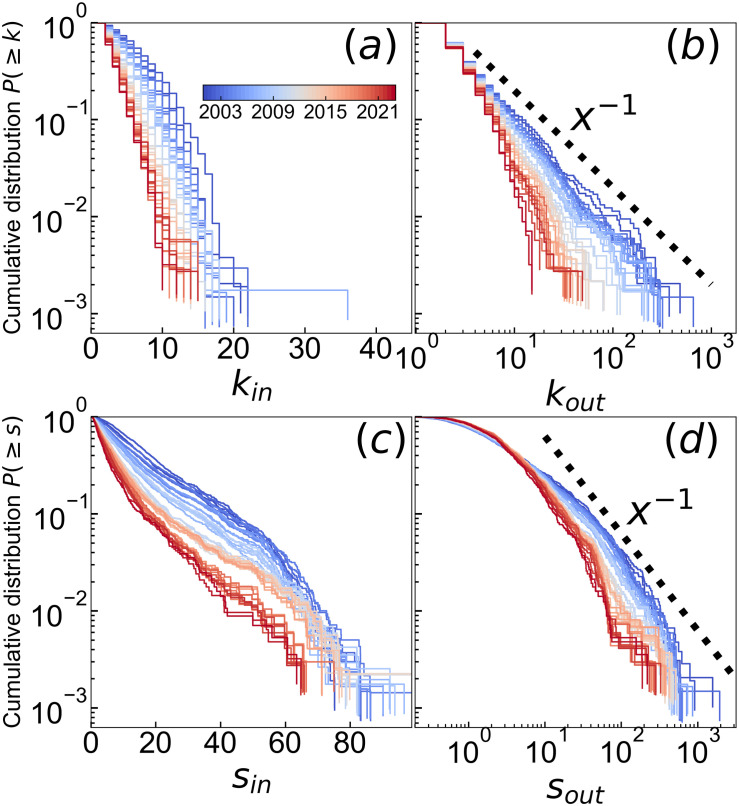
CCDF of (a) in-degree, (b) out-degree, (c) in-strength, and (d) out-strength. The color varies from blue to red with time. The thick black dashed line in (b) and (d) represents a straight line of $x^{-1}$.

Fig 4 presents the complementary cumulative distribution functions (CCDFs) of degree and strength in the SCCN. These functions represent the probability that a value exceeds a given threshold *k* or *s*, denoted by P(≥k and P(≥s, respectively. The slopes of the distributions became steeper over time, which indicates a shift in the underlying distribution. The power-law exponent *α* is defined by the relation


$$P(\geq x)\propto x^{-(\alpha -1)}$$ (10)

for values *x* > *x*_min_. The in-degree and in-strength exhibited linearity in the semi-log plots, which indicates that they followed exponential distributions. In contrast, the out-degree and out-strength appeared linear in the log-log plots, which suggests that they followed power-law distributions. The observation that the out-degree of the SCCN followed a power-law distribution from 2001 to 2010 is consistent with previous findings from 1985 to 2003 [36]. The power-law exponent *α* was estimated using Hill’s method and ranged from 1.68 to 2.69 for the out-degree during 2001–2016 and from 1.63 to 2.47 for the out-strength during 2001–2023. Although the power-law exponent for the out-strength remained relatively stable throughout the period we observed, the out-degree did not follow a power-law distribution in some years because the SCCN focuses on cross-shareholding relationships, which resulted in a smaller cutoff for degree compared to the entire network. Notably, the top five industries in terms of average out-degree in the Full Year of 2023 were banks, pulp & paper, electricity & gas, other financials, and construction, whereas for average out-strength, they were pulp & paper, transportation equipment, electricity & gas, banks, and warehousing & transportation.

### Bow-tie structure

**Fig 5:**
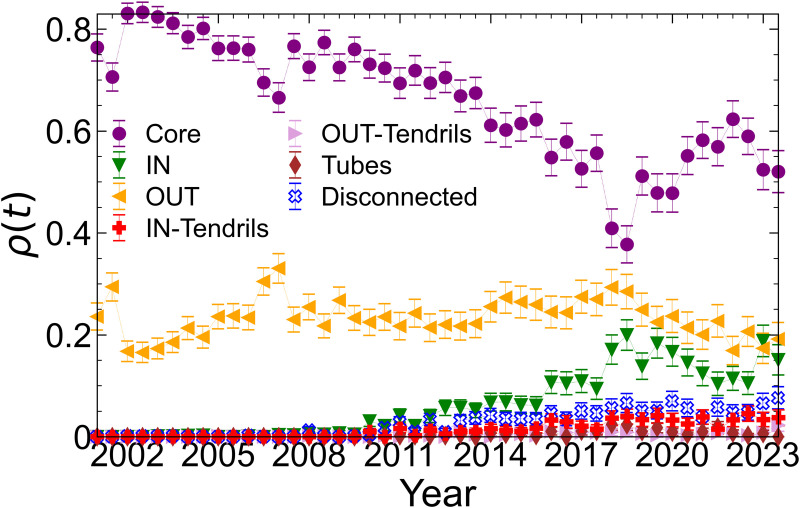
Temporal changes in the ratios of nodes in the bow-tie structure components for the SCCN. Error bars indicate 95% confidence intervals.

Temporal changes in the ratios of the components in the bow-tie structure of the SCCN are shown in Fig 5. Error bars represent 95% confidence intervals. In 2006, a decrease in Core and an increase in OUT was observed, which likely occurred because of an increase in the proportion of foreign shareholders that followed the enforcement of the new Companies Act in May 2006 [42], which resulted in cross-shareholding firms no longer being among the top 30 shareholders.

Around 2010, increases in IN, IN-Tendrils, and Disconnected components were observed. We attribute this finding to the dissolution of cross-shareholding after the introduction of accounting policy changes in 2010, which required listed Japanese companies to disclose the purpose of their shareholdings [43]. Over the entire period, the proportions of IN, IN-Tendrils, and Disconnected nodes increased, whereas the proportion of Core nodes decreased. Increases in IN-Tendrils and Disconnected nodes have also been observed in previous studies [10] which suggests that newly added nodes were incorporated into IN-Tendrils or other networks, rather than into the Core. Since 2015, the Corporate Governance Code has accelerated the dissolution of cross-shareholding which resulted in a decline in the proportion of Core nodes and an increase in the proportion of IN nodes. During the COVID-19 period from 2020 to 2023, the proportion of bow-tie structures remained largely stable despite some fluctuations. The continued decline in the IN component reflected the ongoing trend following the revision of the Corporate Governance Code. In contrast, despite policy initiatives for corporate governance reform such as changes in market classifications [44], the proportion of Core nodes increased.

The most notable changes were a decrease in the Core and increase in IN and Disconnected nodes. In particular, the increase in IN was unique to the SCCN, as this feature was observed across the total network only to a limited extent. Although the size of the SCCN itself decreased, approximately half of the SCCN nodes remained in the SCCN throughout this period. The increase in the IN proportion suggests that some Core nodes may have shifted to IN as cross-shareholding relationships dissolved.

Next, temporal changes in the ratios of the components of the bow-tie for each industry were clustered to identify patterns of similarity. To reduce the impact of noise, we excluded industries with an average of fewer than 15 nodes, resulting in the inclusion of 18 industries in the analysis. Depending on the period, 70% to 80% of the SCCN nodes were included in the clustering analysis. Let $N_{i}^{all}(t)$ denote the total number of companies in industry *i* at time *t* and $N_{i}^{Core}(t)$ denote the number of those in *t*he Core component. We define the Core ratio for each industry as


$$\rho_{i}(t)=\frac{N_{i}^{Core}(t)}{N_{i}^{all}(t)}.$$ (11)

Each industry was represented by a 46-dimensional vector $\left(\rho_{i}(1),\rho_{i}(2),\cdots ,\rho_{i}(46)\right)$. Hierarchical clustering was then performed using the longest-linkage method, with cosine similarity as the distance measure. To emphasize the most salient differences, we set the number of clusters to two. Thus, the Core component was divided into two distinct clusters of industries. Let $n_{i}^{all}(t)$ be the number of companies in cluster *i* at time *t* and $n_{i}^{Core}(t)$ be the number of Core companies within that cluster. The Core ratio at *t*he cluster level is defined as


$$r_{i}(t)=\frac{n_{i}^{Core}(t)}{n_{i}^{all}(t)}.$$ (12)

The same procedure was applied to the OUT and IN components: vectors were constructed for each sector based on the corresponding component ratios, and we then performed hierarchical clustering. This again resulted in two clusters for each component. The hierarchical clustering dendrogram is shown in Supporting Information S1 Fig.

**Fig 6:**
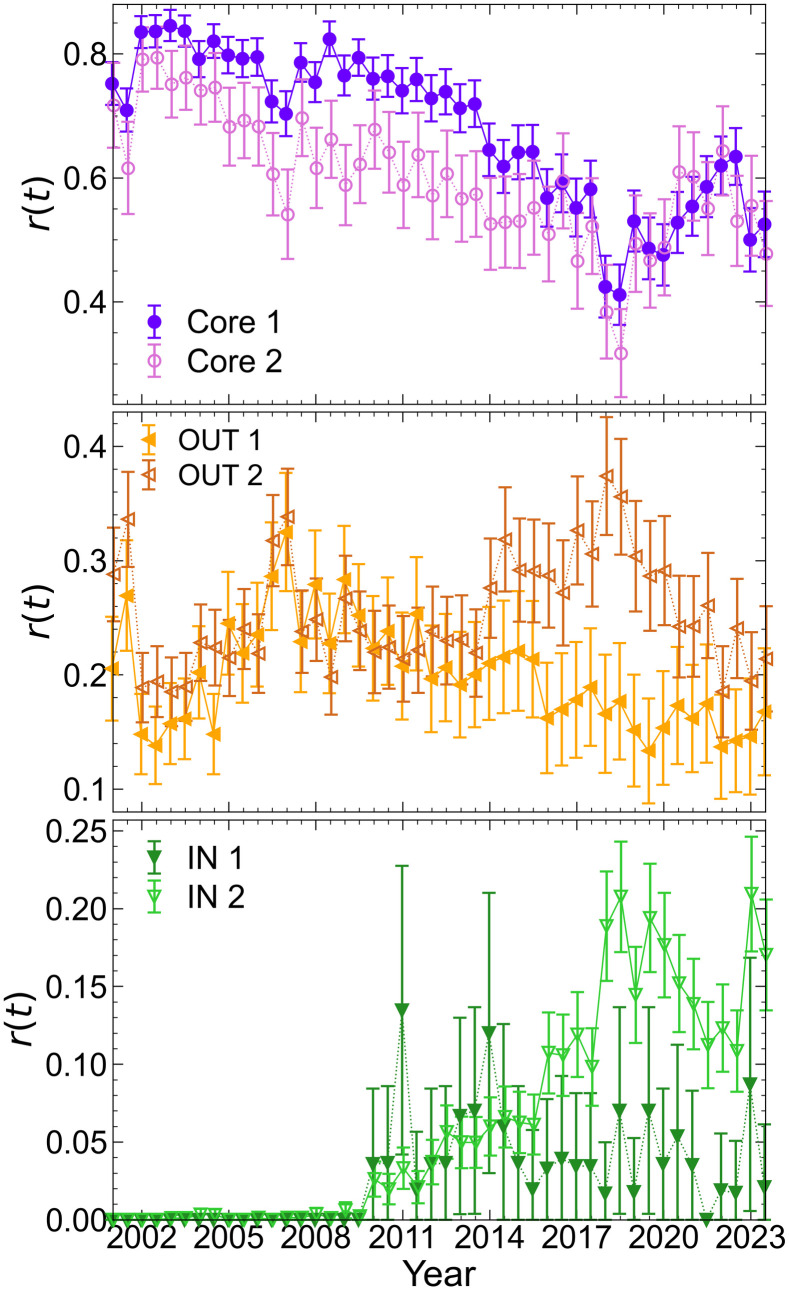
Temporal changes in the ratios of nodes in the Core (top), OUT (middle), and IN (bottom) components of the bow-tie structure shown for two clusters that together include all industries, grouped based on similar temporal patterns. Error bars indicate 95% confidence intervals.

Fig 6 shows the temporal change in the ratio *r*_*i*_(*t*) for the Core, IN, and OUT clusters. Error bars represent 95% confidence intervals. The clusters were characterized by the timing, level, and trends of the changes. The two Core clusters exhibited an overall decline trend; however, the timing and magnitude of this decline differ. The first cluster (Core 1) comprised industries such as banks, real estate, retail trade, iron & steel, construction, and transportation equipment. The second cluster (Core 2) included industries such as information & communication, machinery, services, and textiles & apparels. According to the error bars, the difference between the two clusters was statistically significant from the Interim Year of 2005 through the Interim Year of 2011. After that period, no statistically significant differences were observed between the clusters. Within each cluster, error bars further indicate notable within-group changes. In both clusters, the levels during the Full Year of 2006 through the Interim Year of 2007 differed significantly from those in earlier periods. In Cluster 1, the level subsequently rebounded, followed by another significant decline in Full Year 2014. A further decrease was observed in the Full Year of 2018, but the level recovered thereafter. In Cluster 2, although the ratio reached a minimum value in Interim Year 2007 and increased slightly afterward, the overall level did not exhibit a statistically significant change. A notable decline occurred by Full Year 2015, followed by a recovery similar to that observed in Cluster 1. In the OUT, clusters were characterized by trends after 2016. The first cluster (OUT 1), which declined, comprised banks, real estate, glass & ceramics products, and metal products. The second cluster (OUT 2), which increased in number, included wholesale trade, electrical appliances, construction, foods, transportation equipment, and land transportation. IN clusters were characterized by differences in the degree of increase. Although both clusters had a nearly 0% ratio until 2010, they increased after 2011. The second cluster (IN 2), consisting of information & communication and textiles & apparels, increased in 2015 and then leveled off. Other industries were classified in the first cluster (IN 1), which remained stable after 2011. As indicated by error bars, only a limited number of years exhibited a level that is significantly above zero.

In Japan, banks have traditionally controlled companies through debt and equity [15]. The increase in IN and decrease in OUT for banks suggests that they may have maintained a strong influence, remaining upstream in the hierarchy, even after the dissolution of cross-shareholding. The industries classified in the same cluster as banks across all three components included real estate, transportation equipment, and foods. These industries may occupy similar positions within the hierarchical structure of their ownership network.

### PageRank and HITS distribution

**Fig 7:**
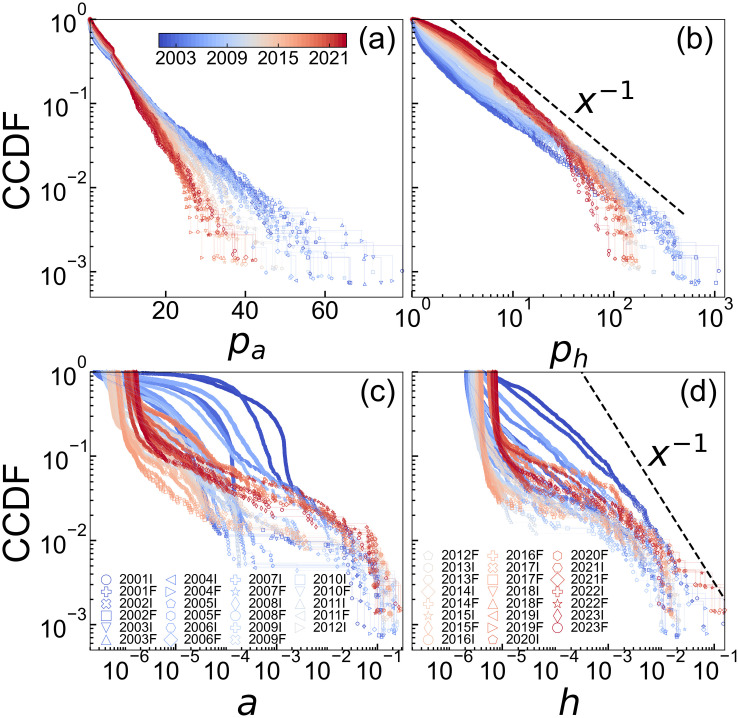
CCDF of (a) p_a_, (b) p_h_, (c) a, (d) h. The color varies from blue to red with time. The thick black dashed line in (b) and (d) represents a straight line of $x^{-1}$.

The complementary distribution functions of *p*_*a*_, *p*_*h*_, *a*, and *h* are shown in Fig 7. The distributions of *p*_*a*_ and *p*_*h*_ were similar to the strength distributions. *p*_*a*_ did not follow a power-law distribution, likely because of the cutoff in the network resulting from the total shareholding percentage not exceeding 100% and constraints on the number of shareholders. In contrast, the power exponent of *p*_*h*_ was stable at approximately 2, which may have been because there was no restriction on the total holdings of other companies. The distributions of *a* and *h* varied in shape depending on the period. The distribution of *a* was characterized by a large concentration of extremely small values which accounted for approximately 90% of the data, whereas a small proportion exhibited extremely large values. This suggested that the probability density function exhibited a bimodal distribution. The distribution of *h* followed a power-law distribution in the early years, specifically from 2001 to 2002. However, after 2010, the distribution of *h* became similar to that of *a*, with a large number of extremely small values and a small number of extremely large values.

**Fig 8:**
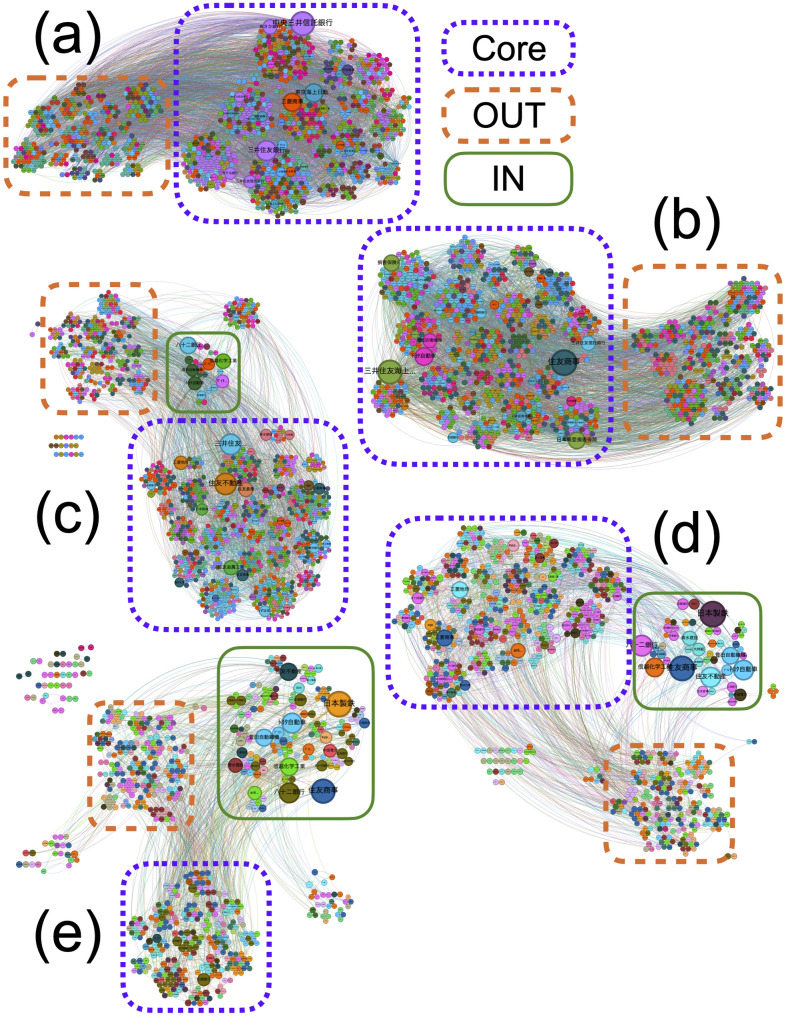
Visualization of the SCCN at five full years: (a) 2001, (b) 2006, (c) 2010, (d) 2018, and (e) 2023. Node positions are arranged according to the bow-tie structure. The Core, OUT, and IN components are enclosed by purple dotted, orange dashed, and green solid lines, respectively. Node colors represent clusters identified using the Louvain method, and node sizes are scaled by the *p*_*h*_ values at each time point.

Fig 8 shows the SCCN for fiscal years 2001, 2006, 2010, 2018, and 2023. The nodes were positioned according to the components of the bow-tie structure and grouped into clusters identified using the Louvain method. The Core, OUT, and IN components are enclosed by purple dotted, orange dashed, and green solid lines, respectively. The node sizes reflected the *p*_*h*_ values at each corresponding time point. Over time, the numbers of nodes and edges in the SCCN decreased. The clusters obtained using the Louvain algorithm did not correspond exactly to the bow-tie components, and multiple communities were observed within each structural region of the bow-tie. The largest nodes were associated with industries such as banking, insurance, construction, and wholesale trade.

### Network structure analysis based on correlations between network measures

We examined correlations among the network measures and demonstrated their temporal invariance. We analyzed the pairwise correlations among six network measures, including *s*_*in*_, *p*_*a*_, *a*, *s*_*out*_, *p*_*h*_, and *h* for the SCCN. As shown in Figs 4 and 7, variables such as *a*, *s*_*out*_, *p*_*h*_, and *h* exhibited highly skewed distributions, with a small number of nodes having extremely large values. Given that Pearson’s correlation coefficient implicitly assumes a normal distribution, we adopted Kendall’s rank correlation coefficient (*τ*) for a robust evaluation without assumptions of the distribution.

**Fig 9:**
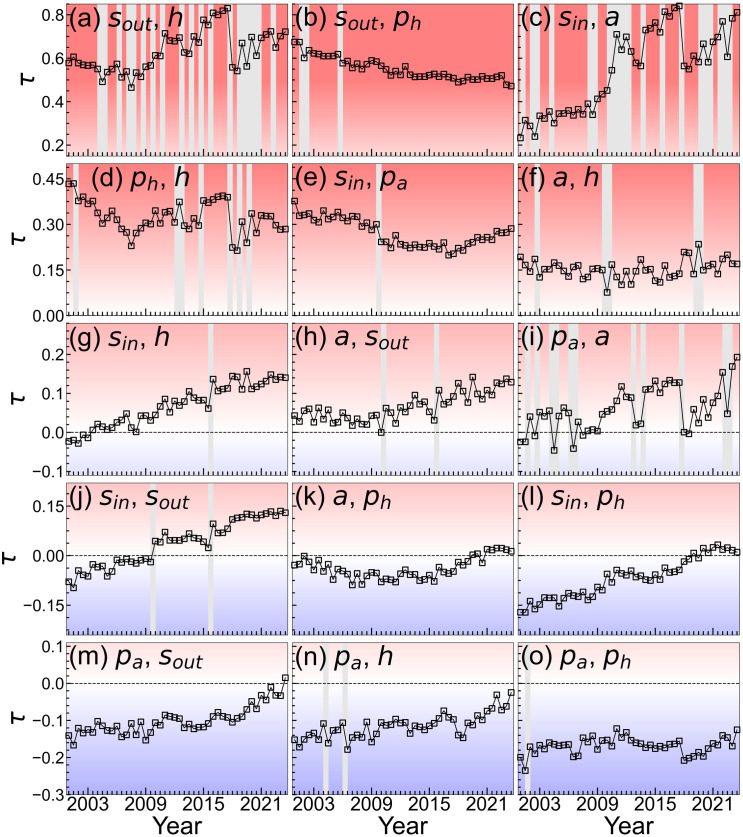
Temporal changes in Kendall’s rank correlation coefficients between pairs of network measures in the SCCN. The subfigures are shown in descending order based on the average Kendall’s rank correlation coefficient (*τ*) over time. Gray shading represents the consecutive time points at which the 95% bootstrap confidence interval of the difference in Kendall’s *τ* excluded zero, indicating statistical significance at the 5% level. For example, panel (a) shows the temporal variation in the correlation between *s*_*out*_ and ***h*.**

Temporal changes in Kendall’s *τ* among the network measures are shown in Fig 9. To examine whether Kendall’s *τ* between the measures of centrality varied significantly over time, we conducted a bootstrap hypothesis test for each consecutive time pair (*t*, *t* + 1). For each test, we generated *B* = 10000 bootstrap replications of the difference ($\Delta =\tau_{t+1}-\tau_{t}$) by resampling (*x*,*y*) pairs with replacement from each time period. We then constructed a 95% percentile-based confidence interval for *Δ* from the resulting bootstrap distribution to evaluate whether the null hypothesis of Δ=0 could be rejected. This procedure was applied to all pairs of centrality measures across consecutive time periods. The gray shading in Fig 9 indicates the consecutive time points for which the 95% confidence interval for *Δ* excluded zero, indicating statistical significance at the 5% level. Panels (a)–(o) of Fig 9 are arranged in descending order of average correlation over time. The correlation structure among the measures of centrality remained largely stable over time despite a gradual decrease in the size of the network.

Strong positive correlations were observed between *s*_*out*_ and *h*, *s*_*out*_ and *p*_*h*_, and *s*_*in*_ and *a* (Fig 9(a), 9(b), 9(c)). While the signs of the correlations remained unchanged, the correlations between *s*_*out*_ and *h*, and between *s*_*in*_ and *a*, differed significantly between interim and annual reporting periods. This likely occurred because *h* and *a* are sensitive to changes in degree [37], and the disclosure criteria differ between interim and annual reports. Some correlations among the measures of centrality arise naturally from their mathematical definitions [45]. Consistent with previous studies, networks with many reciprocal links tended to exhibit strong correlations between strength and PageRank. In our study, the strong correlations between *s*_*out*_ and *p*_*h*_ and between *s*_*in*_ and *p*_*a*_ reflect the presence of numerous reciprocal shareholding relationships. Relatively strong positive correlations were also observed between *p*_*h*_ and *h* and between *s*_*in*_ and *p*_*a*_ (Fig 9(d)(e)). A weak positive correlation was observed between *a* and *h* (Fig 9 (f)). These results were consistent with trends observed in corporate transaction networks [1] and other networks such as the World Wide Web [46]. The correlation between *p*_*a*_ and *a* was weaker than that between *p*_*h*_ and *h* (Fig 9(d)(i)). This, along with the weaker correlation between *s*_*in*_ and *p*_*a*_ compared to *s*_*out*_ and *p*_*h*_ (Fig 9(b)(e)), indicated an asymmetry between *s*_*in*_ and *s*_*out*_ at the node level. In contrast to previous research, which reported a weak positive correlation between *p*_*a*_ and *a*[1], our analysis showed a trend toward no correlation (Fig 9(i)). This suggests that within a cross-shareholding network, having shareholders who invest in many firms does not necessarily correlate with susceptibility to influence. Given that firms that invest in a large number of other companies tend to be banks [25], and banks often invest across a wide range of companies—from large enterprises to small and medium-sized firms—this result is intuitive. Furthermore, *p*_*a*_ and *p*_*h*_ exhibited a negative correlation (Fig 9(o)) which suggests the existence of a ranking structure among firms in terms of influence: some firms were highly influential and less susceptible to influence, whereas others were less influential and more susceptible.

The correlation between *s*_*in*_ and *s*_*out*_ was an exception to the temporal invariance because it changed over time (Fig 9(j)). It changed significantly between 2009–2010 and 2015–2016 which correspond to the global financial crisis and the revision of the Corporate Governance Code, respectively. Although the correlation was weakly negative from 2001 to 2005, it became weakly positive after 2016. This implies that companies with more outgoing shareholdings have become more likely to receive shareholdings. This result suggests that during these periods firms that tended to hold shares were also more likely to be held by others, which indicates that cross-shareholding relationships became more entrenched. As cross-shareholding relationships were gradually dissolved, the remaining network increasingly consisted of firms that maintained strong mutual shareholding ties.

### Characterizing industries based on correlations between network measures

**Fig 10:**
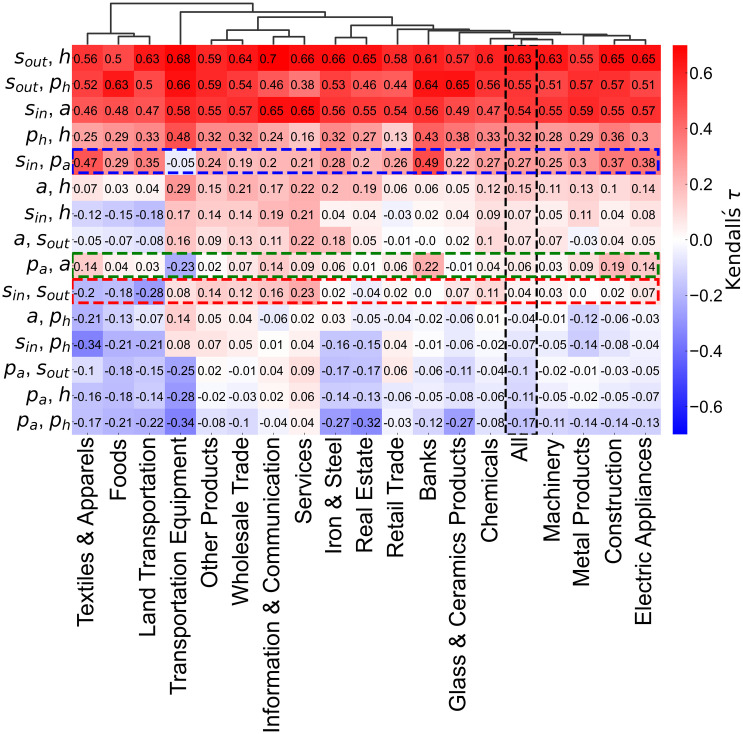
Kendall’s rank correlation coefficients between pairs of network measures by industry. The vertical axis represents pairs of network measures, and the horizontal axis represents industries. The heatmap shows the time-averaged Kendall’s rank correlation coefficients for each industry. The dendrogram is the result of hierarchical clustering using Euclidean distance and Ward’s method, where each industry was characterized by a 15-dimensional vector of Kendall’s rank correlation coefficients across all measure pairs.

We analyzed whether the correlations among network measures varied by industry. Fig 10 presents a heatmap of the time-averaged Kendall’s rank correlation coefficients between pairs of network measures, calculated for nodes within each industry. The column labeled “All” shows the time-averaged correlation coefficients obtained from all nodes across the SCCN (black dashed-line rectangle in Fig 10). The dendrogram was derived from a 15-dimensional feature vector $\left(\tau_{i}^{1},\tau_{i}^{2},\ldots ,\tau_{i}^{15}\right)$ for each industry *i*, where $\tau_{i}^{j}$ denotes Kendall’s rank correlation coefficient for the network measure pair *j* within industry *i*. Clustering was performed using the Euclidean distance and Ward’s method. For most pairs of measures, the correlation patterns of the individual industries closely resembled those of the overall network. However, notable deviations were observed in industries such as transportation equipment, textiles & apparels, foods, and land transportation.

For example, the pair *s*_*in*_ and *p*_*a*_ was uncorrelated only in the transportation equipment industry while exhibiting a positive correlation in the overall network and other industries (blue dashed-line rectangle in Fig 10). The absence of a correlation in this sector can be attributed to its unique structure: large corporations such as Denso and Toyota Industries—suppliers to Toyota Motor Corporation—and major automobile manufacturers such as SUBARU are heavily owned by Toyota. However, these companies may also own shares in Toyota or invest in firms outside the Toyota group which results in high *s*_*in*_ without a correspondingly high *p*_*a*_. While the pair *a* and *p*_*a*_ exhibited either no correlation or a weak positive correlation in general, the transportation equipment industry exhibited a negative correlation (green dashed-line rectangle in Fig 10). A positive correlation implied that firms sharing common shareholders tended to be more susceptible to external influences (or vice versa), whereas a negative correlation suggested that having similar shareholders may have reduced such susceptibility. In hierarchical industries such as transportation equipment, where large corporations such as Toyota serve as major shareholders of many affiliated companies, firms with high *a* may still have low *p*_*a*_ because they are unlikely to be influenced by entities other than Toyota.

The pair *s*_*in*_ and *s*_*out*_ had no significant correlation across all the nodes from the SCCN. However, textiles & apparels, foods, and land transportation had negative correlations, whereas industries such as other products, wholesale trade, information & communication, services, and chemicals exhibited weak positive correlations (red dashed-line rectangle in Fig 10). A negative correlation indicated non-reciprocal shareholding relationships, in which firms that held more shares were less likely to be held by others (or vice versa). In contrast, a positive correlation suggested reciprocal relationships in which firms that invested more were also more likely to be invested in. Industries with negative correlations, such as textiles & apparels, foods, and land transportation, are typically B-to-C businesses. Their capital or business relationships tend to be one-directional and are often influenced by corporate group structures such as conglomerates and their subsidiaries, trading companies and their affiliates, or national and regional firms. In contrast, industries with positive correlations such as other products, wholesale trade, information & communication, services, and chemicals tend to operate in B-to-B domains, where cross-shareholding is often accompanied by mutual business complementarities.

### Characterizing industries based on percentile ranks of network measures

We analyzed the temporal variations in network metrics across industries. To investigate the temporal dependence of the ranking order of these metrics, we examined their percentile ranks. The percentile rank (PR) of a given value represents the percentage of smaller data points and is defined as


$$PR=\frac{B}{N},$$ (13)

where *B* denotes the number of values smaller than the given value and *N* is the total number of observations. The percentile rank of the network metric *i* at time *t* obtained by averaging the percentile ranks across the nodes in industry *j* is denoted by $PR_{i}^{j}(t)$.

**Fig 11:**
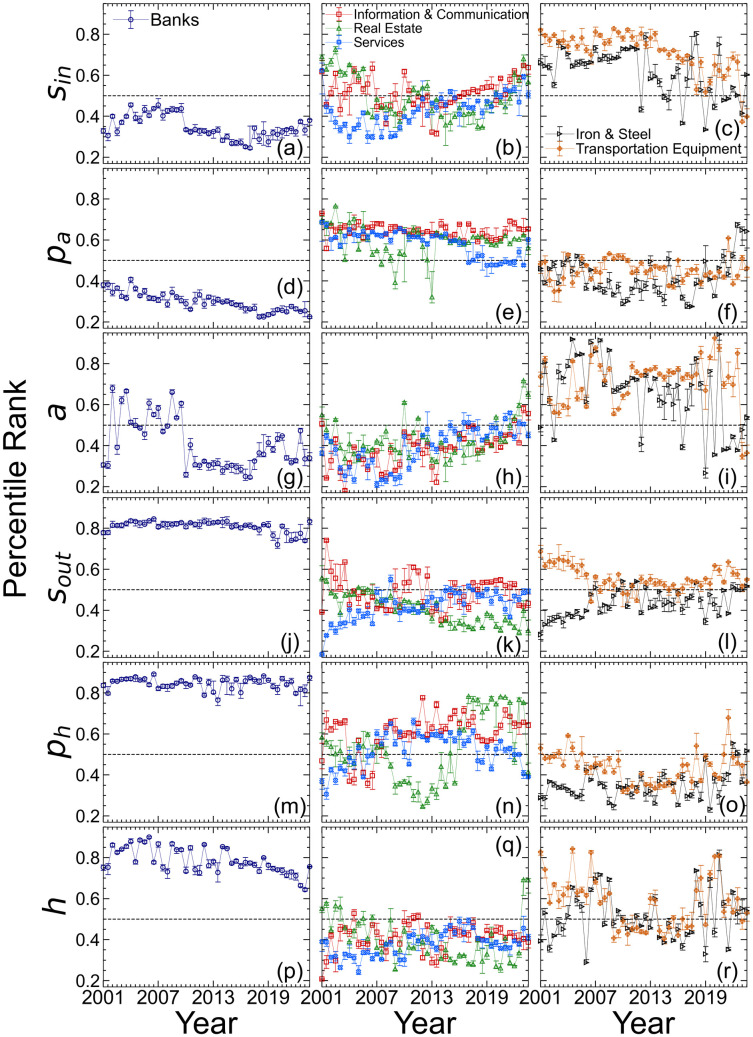
Temporal changes in the percentile ranks of s_in_, p_a_, a, s_out_, p_h_, and h for three representative clusters. These clusters are selected examples from the seven clusters obtained via hierarchical clustering. Each subplot displays the time series of the average percentile rank for a specific network measure within one of these representative clusters. Industries are grouped in columns within each subplot based on the clustering result obtained using Euclidean distance and Ward’s method. Each industry is represented by a 276-dimensional vector comprising the percentile ranks of six network measures, *s*_*in*_, *p*_*a*_, *a*, *s*_*out*_, *p*_*h*_, and *h*, across 46 time points. Markers indicate the average percentile rank at each time point, and error bars represent the 45th to 55th percentile range. The left, center, and right subplots correspond to clusters respectively composed of banks, then information & communication, real estate, and services, and finally iron & steel and transportation equipment.

Each industry *j* is characterized by a 276-dimensional vector constructed from the 46 time-point percentile ranks $PR_{i}^{j}(t)$ of the six network measures *i*, specifically $\left({\mathrm{PR}}_{1}^{j}(1),{\mathrm{PR}}_{1}^{j}(2),\ldots ,{\mathrm{PR}}_{1}^{j}(46),{\mathrm{PR}}_{2}^{j}(1),\ldots ,{\mathrm{PR}}_{2}^{j}(46),\ldots ,{\mathrm{PR}}_{6}^{j}(1),\ldots ,{\mathrm{PR}}_{6}^{j}(46)\right)$. We performed hierarchical clustering using Euclidean distance and Ward’s method and grouped the industries into seven clusters. Although some fluctuations were observed, the relative rankings of industries classified as high, medium, or low have remained largely stable over time. Fig 11 shows the temporal changes in the percentile rankings of *s*_*in*_, *p*_*a*_, *a*, *s*_*out*_, *p*_*h*_, and *h* for three of the seven identified clusters. Each marker represents the percentile ranking at a given time point, and the error bars indicate the range from the 45th to 55th percentile. Detailed temporal trends for all industries are provided in the Supporting Information S2 and S3 Figs.

**Fig 12:**
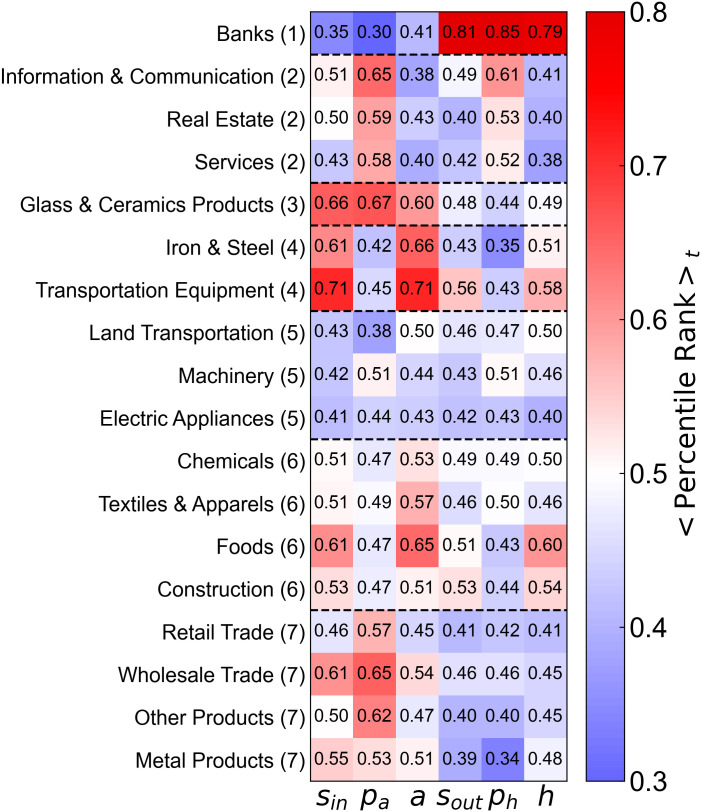
Time averages of the percentile ranks of s_in_, p_a_, a, s_out_, p_h_, and h by each industry. Each cell shows the time-averaged percentile rank of a network metric for a given industry. Rows correspond to industries, and columns to network metrics. The heatmap color scale ranges from blue (low percentile rank) to red (high percentile rank). The number following each industry name indicates the cluster to which the industry belongs based on the similarity of temporal variations in percentile ranks. These clusters were obtained by applying hierarchical clustering to 276-dimensional vectors (six network measures across 46 time points) for each industry, as visualized in S2 and S3 Figs. The black dashed lines in the heatmap denote the boundaries between clusters.

Cluster 1 (as shown in the left column of Fig 11, which shows the clusters selected from the full set of results shown in Fig 12) consisted solely of banks, which consistently ranked high in *s*_*out*_, *p*_*h*_, and *h* across all periods while ranking consistently low in *s*_*in*_ and *p*_*a*_. The rank of *a* remained at a mid-range level until around 2010 and declined thereafter, possibly because of post-crisis disclosure regulations designed to reduce banks’ cross-shareholding. A high rank for *p*_*h*_ indicates the strength of a bank’s influence within the network. This result contrasts with that in Germany, where regulations diminished banks’ influence on cross-shareholding relationships [47]. Despite the unwinding of cross-shareholding ties, the high *h* ranking suggests that banks continued to hold shares in many companies with cross-shareholding relationships. The finding that banks function as hubs was consistent with the observations in the Netherlands [48]. However, the downward trend in rankings from 2015 implied a reduced capacity to exert influence across the network. Cluster 2 (shown in the center column of Fig 11) included industries such as information & communication, real estate, and services. These industries ranked low in *a*, *s*_*out*_, *a*nd *h* but tended to rank high in *p*_*a*_ and *p*_*h*_. This suggests that these industries were highly susce*p*tible to influence and had considerable influence within the network. Cluster 4 (right column of Fig 11) included iron & steel and transportation equipment. These industries ranked high in *s*_*in*_ and *a* but low in *p*_*a*_ *a*nd *p*_*h*_. This indicates strong internal cross-shareholding with many shared shareholders but a relatively limited influence at the broader network level. As shown in Fig 10, trans*p*ortation equi*p*ment has a negative correlation between *p*_*a*_ and *a*. The high *a a*nd *s*_*in*_ but low *p*_*a*_ values suggest that firms in these industries formed dense internal cross-shareholding networks (e.g., keiretsu) which reduced external influence.

The industries exhibited stable percentile ranks over time for most centrality measures. For example, banks consistently ranked low in *p*_*a*_ and *s*_*in*_ but high in *p*_*h*_ and *h*, which indicates strong outbound influence but limited inbound exposure. Industries such as information & communication and real estate showed the opposite pattern, which reflects their rising susceptibility to influence.

Fig 12 shows the industry-wise time-averaged percentile ranks for the six network measures *s*_*in*_, *p*_*a*_, *a*, *s*_*in*_, *p*_*h*_, and *h* across the 18 industries. The number following the name of each industry indicates the cluster to which the industry belongs. Cluster 1 includes banks; this cluster was characterized by low percentile ranks in *s*_*in*_, *p*_*a*_, and *a*, and high percentile ranks in *s*_*out*_, *p*_*h*_, and *h*. Cluster 2 includes information & communication, real estate, and services; this cluster exhibited high percentile ranks in *p*_*a*_ and *p*_*h*_ and low ranks in *a a*nd *h*. Cluster 3 includes glass & ceramics *p*roducts; this cluster showed high percentile ranks in *s*_*in*_, *p*_*a*_, and *a* and low r*a*nks in *s*_*out*_, *p*_*h*_, and *h*. Cluster 4 consists of iron & steel and trans*p*ortation equi*p*ment; this cluster was marked by high percentile ranks in *s*_*in*_ and *a* and low ranks in *p*_*a*_ and *p*_*h*_. This indicated strong internal cross-shareholding with many shared shareholders but a relatively limited influence at the broader network level. Transportation equi*p*ment showed a negative correlation between *p*_*a*_ and *a*. The high *a* and *s*_*in*_ but low *p*_*a*_ values suggest that firms in these industries formed dense internal cross-shareholding networks (e.g., keiretsu) that reduced external influence. Cluster 5 includes land transportation, machinery, and electric a*pp*liances; this cluster was characterized by mid- to low-level percentile ranks across all six metrics. Cluster 6 comprised chemicals, textiles & apparels, foods, and construction; this cluster exhibited moderate percentile ranks for all six indicators. This group exhibited patterns similar to those of Cluster 4 but generally ranked higher across most network measures. In particular, foods exhibited high average rankings for both *a* and *h*, likely because of its cross-shareholding ties with m*a*jor conglomerates and trading companies. Finally, Cluster 7 comprised retail trade, wholesale trade, other products, and metal products; this cluster showed high percentile ranks for *p*_*a*_ and low ranks for *s*_*out*_, *p*_*h*_, and *h*. For retail trade, this may reflect significant investments from large wholesale traders, resulting in a high susceptibility to influence. A similar trend was inferred for the other product sectors.

## Discussion

In this study, we investigated the structure and long-term evolution of cross-shareholding networks among all of the listed Japanese companies from the perspectives of econophysics and network science. By employing bow-tie decomposition, measures of centrality derived from the PageRank and HITS algorithms, and analyzing the correlations among these measures, we identified characteristic hierarchical patterns and industry-level differences in the shareholding network.

The analysis of the bow-tie structure revealed a temporal decline in the proportion of nodes belonging to the Core component, along with an increase in the proportions of IN and Disconnected nodes. This trend reflects the gradual unwinding of cross-shareholding ties over time. However, the persistence of the SCC-based network suggests that such ownership relationships have not been entirely dissolved. Moreover, newly added companies have not been integrated into the core structure but rather remain in peripheral positions such as IN-Tendrils or Disconnected components.

We found that the correlation structure among the centrality measures based on PageRank and HITS remained relatively stable from 2001 to 2023. This persistence may be attributed to the enduring features of Japanese corporate governance. Although policy pressures have reduced the overall volume of cross-shareholdings, the fundamental emphasis on long-term stability and stable affiliated relationships, including keiretsu ties, appears to have remained structurally preserved. This temporal stability suggests that the underlying structure of the shareholding network is robust over time and may reflect structural regularities analogous to those observed in physical systems.

The industry-level analysis further revealed that while the overall correlation patterns were similar across most industries, some sectors deviated from this general trend. For example, B-to-C sectors such as textiles & apparels, foods, and land transportation exhibited negative correlations between incoming and outgoing links which suggests asymmetric shareholding relationships. In contrast, B-to-B sectors such as wholesale trade and chemicals exhibited weakly positive correlations which implies more reciprocal cross-shareholding ties. These differences likely reflect the distinct capital structures and business strategies inherent in each industry. Specifically, these B-to-C industries are typically supplied by upstream B-to-B firms but sell to consumers, creating a directional flow of capital and goods that may reduce the formation of reciprocal trading ties commonly observed in purely B-to-B sectors. Furthermore, the unique correlation pattern in the transportation equipment sector reflects the vertical keiretsu structure (e.g., the Toyota group), where suppliers are tightly integrated through ownership but are relatively insulated from broader external market influences compared to other sectors.

Altogether, our findings highlight that cross-shareholding relationships are not merely financial linkages but also form structural hierarchies shaped by industry-specific characteristics and long-term institutional dynamics. The persistence of certain structural features across time suggests the presence of robust organizational patterns, which may be of interest in network science and econophysics. They can also provide guidelines for policymakers concerned with corporate governance and market transparency.

Our finding of temporal invariance in centrality correlations despite major events such as the 2010 accounting reforms, the 2015 Corporate Governance Code and the COVID-19 pandemic suggests that certain structural properties of the cross-shareholding network are highly persistent. This indicates that traditional firm-level or short-term monitoring may miss deeper systemic patterns in ownership. We recommend that regulators implement periodic network-based monitoring systems to identify structurally influential firms or clusters that are resilient to policy and economic shocks, especially in critical sectors such as banks, information & communication, and real estate. Furthermore, the persistently high influence (e.g., high *p*_*h*_) of banks, along with a declining hub score, im*p*lies a shift in how influence is exercised in ownership networks. Simultaneously, industries such as information & communication and real estate have become increasingly influential in terms of susceptibility and impact. These sectoral transitions suggest that emerging industries with rising centrality should receive more focused governance scrutiny and oversight because their growing influence could shape market behavior and systemic risk in new ways. Policymakers should consider targeted corporate governance reinforcement in these sectors to ensure transparent and accountable ownership practices.

This study involves several limitations, the first of which relates to data constraints. The data cutoff of 30 major shareholders underestimated the number of nodes and edges compared with the actual network. The second limitation is related to the choices made while the network is constructed. Individuals, governments, and funds were excluded to focus on corporate cross-shareholding structures, meaning that their influences were not considered. Focusing on the local structure of a network may introduce a bias when evaluating its influence using PageRank. However, the data used in this study covered all of the listed Japanese companies, including large corporations. Because less important nodes were removed owing to the cutoff, the impact of noise was reduced which resulted in robust findings regarding temporal changes in bow-tie structure, PageRank, and similarities between industries. A final limitation concerns the scope of the study, which was confined to a single national context, Japan. Thus, the generalizability of the findings to other countries may be limited.

Future studies could incorporate the financial and governance-related attributes of firms to further explore the underlying drivers and consequences of cross-shareholding. A comparative analysis across countries and markets would also aid in elucidating the institutional and cultural factors that shape ownership network structures. This would involve further decomposition of the bow-tie structure to clarify the positional relationships among the nodes. Investigating motifs within cross-shareholding relationships may reveal the underlying patterns [49]. Structural factors that cause variations in the centrality indices should also be examined. Recent studies have used rank-1 approximation to identify structural drivers of empirical PageRank distributions [50,51]. Extending such approaches to weighted and directed networks and applying them to PageRank, authority and hub scores would provide deeper insights into network structure. Analyzing fluctuations in PageRank at the node level or at a more detailed industry level could also enrich the discussion presented in this study. Furthermore, exploring the relationships between PageRank or authority and hub scores and corporate finance may reveal underlying mechanisms.

## Supporting information

S1 Fig Dendrograms from hierarchical clustering of 18 industries based on the temporal ratios of companies in the Core, OUT, and IN components of the SCCN. (TIF)

S2 Fig Temporal changes in the percentile ranks of s_in_, p_a_, and a by industry. (TIF)

S3 Fig Temporal changes in the percentile ranks of s_out_, p _h, and_ h by industry. (TIF)
