# Supplementary figures and images for: Temporal invariance of centrality correlations and hierarchical topology in evolving cross-shareholding networks in Japan (2001–2023)
Source: PLoS One. 2026 Mar 25;21(3):e0331561. doi: 10.1371/journal.pone.0331561 (PMC13020756; doi:10.1371/journal.pone.0331561)

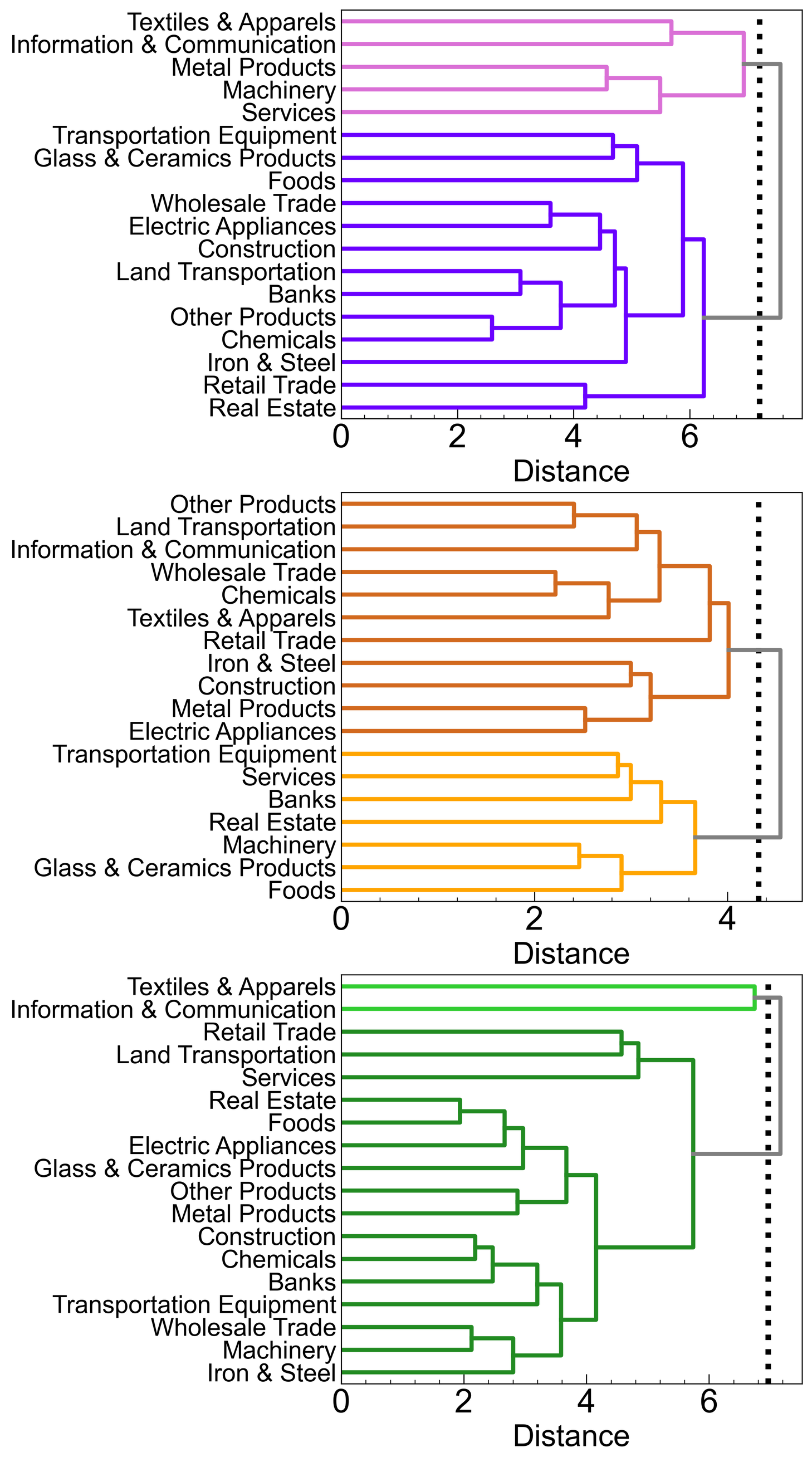

Supplement: S1 Fig — (TIF) [file pone.0331561.s001.tif]

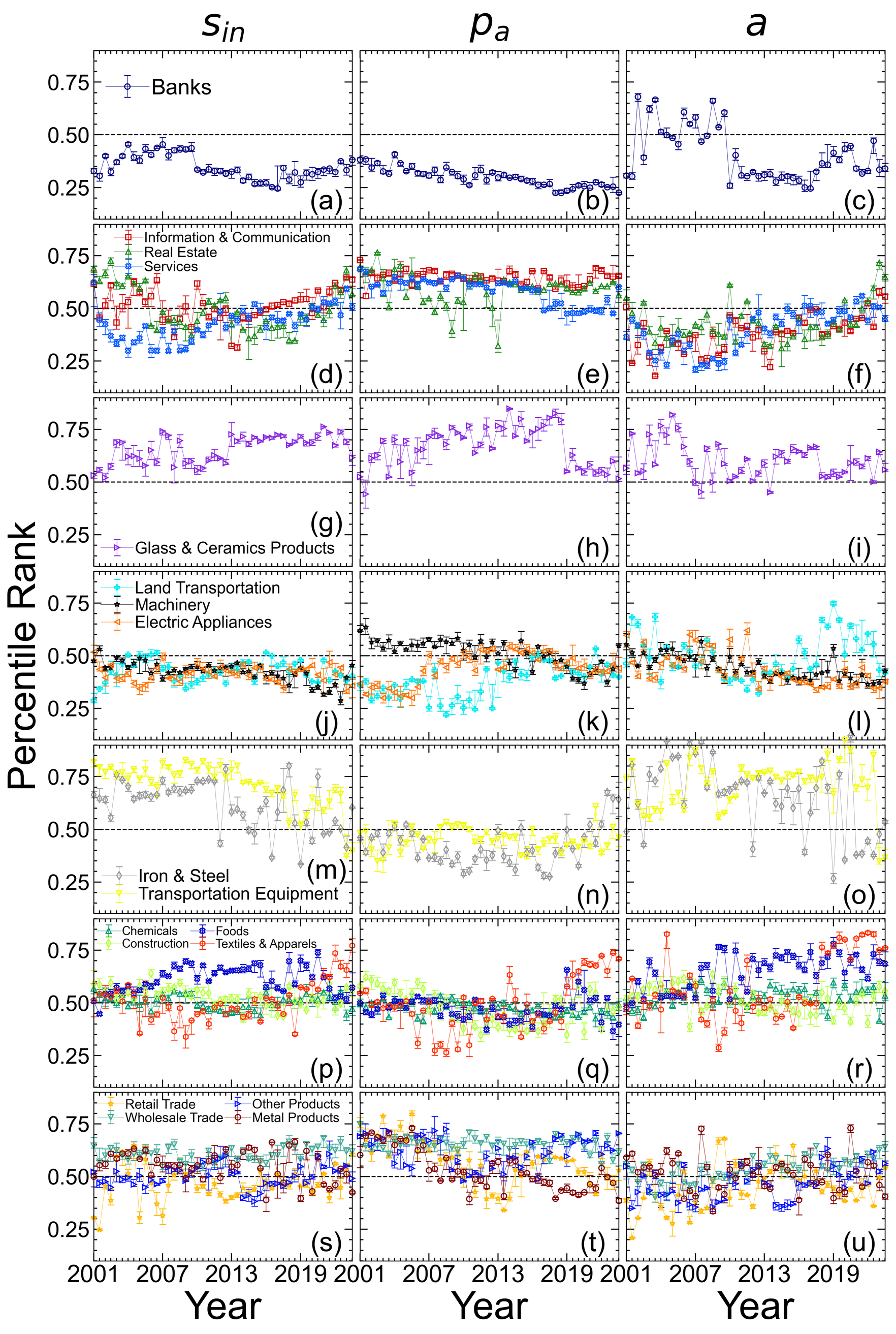

Supplement: S2 Fig — (TIF) [file pone.0331561.s002.tif]

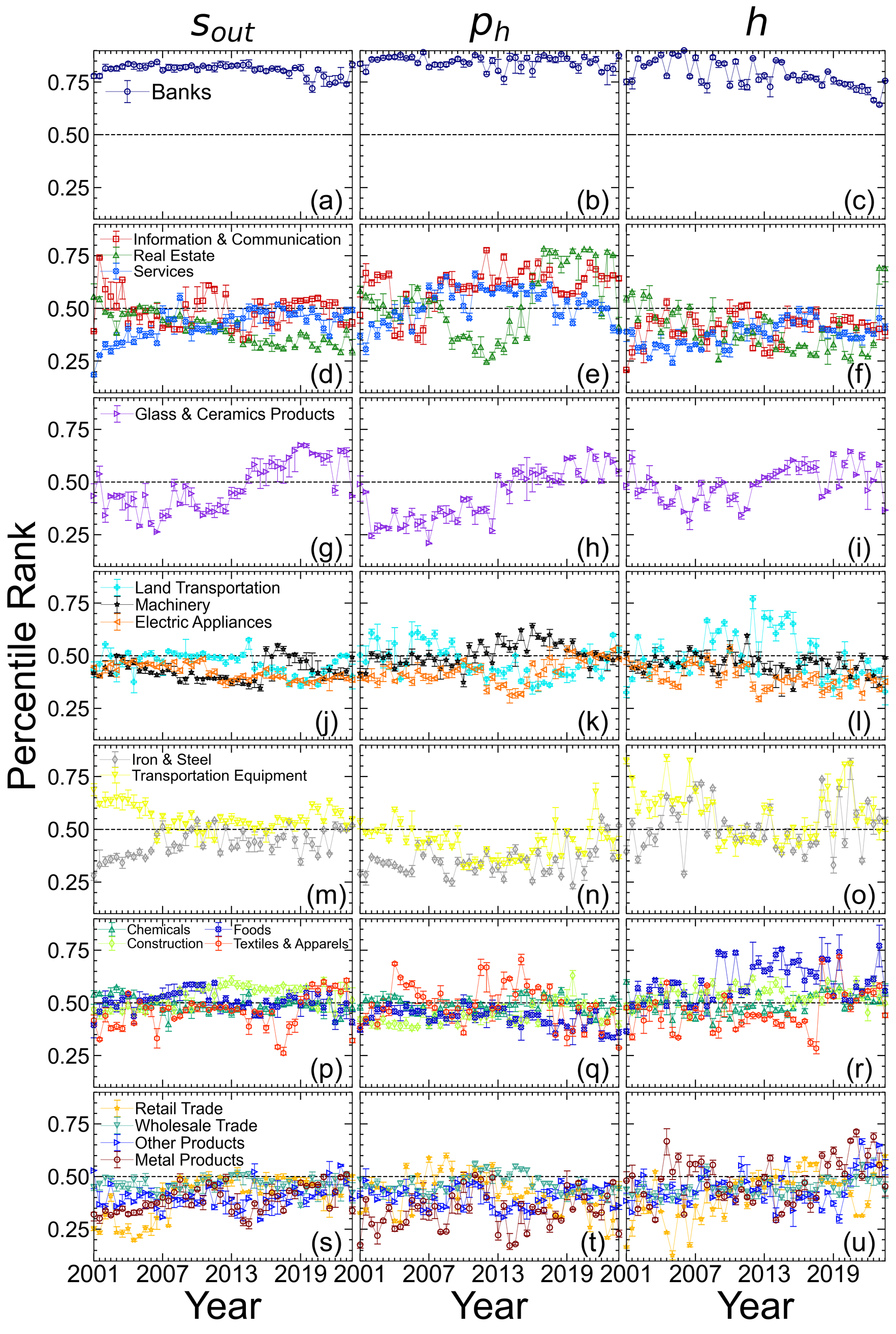

Supplement: S3 Fig — (TIF) [file pone.0331561.s003.tif]
